# Optimization of the “Perth CT” Protocol for Preoperative Planning and Postoperative Evaluation in Total Knee Arthroplasty

**DOI:** 10.3390/medicina60010098

**Published:** 2024-01-05

**Authors:** Milica Stojadinović, Dragan Mašulović, Marko Kadija, Darko Milovanović, Nataša Milić, Ksenija Marković, Olivera Ciraj-Bjelac

**Affiliations:** 1 Center for Radiology, University Clinical Center of Serbia, Pasterova 2, 11000 Belgrade, Serbia; draganmasulovic@yahoo.com; 2 Faculty of Medicine, University of Belgrade, 11000 Belgrade, Serbia; kadija.marko@gmail.com (M.K.); natasa.milic@med.bg.ac.rs (N.M.); 3 Clinic for Orthopedic Surgery and Traumatology, University Clinical Center of Serbia, Pasterova 2, 11000 Belgrade, Serbia; 4 Institute for Medical Statistic and Informatics, University Clinical Center of Serbia, Pasterova 2, 11000 Belgrade, Serbia; xeniakm3@gmail.com; 5 Department of Internal Medicine, Division of Nephrology and Hypertension, Mayo Clinic, Rochester, MN 55905, USA; 6 Vinca Institute of Nuclear Sciences—National Institute of the Republic of Serbia, 11000 Belgrade, Serbia; ociraj@vinca.rs; 7 Faculty of Electrical Engineering, University of Belgrade, 11000 Belgrade, Serbia

**Keywords:** total knee arthroplasty, Perth CT protocol, optimization

## Abstract

*Background and Objectives*: Total knee arthroplasty (TKA) has become the treatment of choice for advanced osteoarthritis. The aim of this paper was to show the possibilities of optimizing the Perth CT protocol, which is highly effective for preoperative planning and postoperative assessment of alignment. *Materials and Methods*: The cross-sectional study comprised 16 patients for preoperative planning or postoperative evaluation of TKA. All patients were examined with the standard and optimized Perth CT protocol using advance techniques, including automatic exposure control (AEC), iterative image reconstruction (IR), as well as a single-energy projection-based metal artifact reduction algorithm for eliminating prosthesis artifacts. The effective radiation dose (E) was determined based on the dose report. Imaging quality is determined according to subjective and objective (values of signal to noise ratio (SdNR) and figure of merit (FOM)) criteria. *Results*: The effective radiation dose with the optimized protocol was significantly lower compared to the standard protocol (*p* < 0.001), while in patients with the knee prosthesis, E increased significantly less with the optimized protocol compared to the standard protocol. No significant difference was observed in the subjective evaluation of image quality between protocols (*p* > 0.05). Analyzing the objective criteria for image quality optimized protocols resulted in lower SdNR values and higher FOM values. No significant difference of image quality was determined using the SdNR and FOM as per the specified protocols and parts of extremities, and for the presence of prothesis. *Conclusions*: Retrospecting the ALARA (‘As Low As Reasonably Achievable’) principles, it is possible to optimize the Perth CT protocol by reducing the kV and mAs values and by changing the collimation and increasing the pitch factor. Advanced IR techniques were used in both protocols, and AEC was used in the optimized protocol. The effective dose of radiation can be reduced five times, and the image quality will be satisfactory.

## 1. Introduction

Total knee arthroplasty (TKA) has become the treatment of choice for advanced osteoarthritis [1,2]. Kurtz et al. [3] projected a 673% increase in TKA in the United States (US) between 2005 and 2030. In addition, Inacio et al. [4] predicted that the volume of TKA procedures in the US will increase by 143% and 855% from 2012 to 2050, based on conservative and exponential growth assumptions, respectively.

Accurate positioning of the component and proper adjustment of soft tissue are recognized as crucial factors in achieving a successful knee arthroplasty [5,6]. Incorrect alignment can lead to abnormal wear [7], premature mechanical loosening [8] of the components, and patellofemoral problems [9]. There is a clear correlation between the precision of implant placement and the long-term sustainability [10]. Assessing alignment poses a significant difficulty. Conventional radiography, long-leg films, and CT scanograms were all deemed insufficient and hence rejected [11,12]. Consequently, the “Perth CT Protocol” was created as outlined in [13]. The method is highly effective for preoperative planning and postoperative assessment of alignment, particularly rotational alignment [14]. The Perth CT protocol demonstrates excellent intra-observer reliability and good to excellent inter-observer reliability [1]. The procedure involves conducting CT imaging of the relevant limb, starting from the roof of the acetabulum and extending to the arch of the talus [15]. The limb should be in a supine posture, with the leg in a neutral alignment, and maximal knee extension. Postprocedurally, by reformation in the coronal, sagittal, and axial planes, seven characteristics of alignment as well as the mechanical and anatomical axis of the leg are determined. For the femoral component, varus/valgus, flexion/extension, and rotation are determined, and for the tibial component, varus/valgus, posterior slope, and rotation are determined. Postprocedurally, both femoral and tibial joint mismatch are also assessed.

Any form of imaging that involves the use of ionizing radiation poses a potential risk of genetic damage and malignancy. The average annual radiation exposure per person is estimated to be 2.7 mSv in the United Kingdom [16]. In comparison, the long-leg AP standing radiograph delivers a dose of around 0.7 mSv [17], the Perth protocol for lower-limb CT scans delivers a dose of around 2.7 mSv [13], and the Imperial protocol for lower-limb CT scans delivers a dose of around 0.761 mSv [18]. Henckel and colleagues [18] developed the low-dose Imperial CT protocol, which administers a radiation dosage ranging from 0.53 to 0.84 mSv. This dose is nearly equivalent to that of X-rays.

The AURORA protocol, developed by Wakelin et al. [19] is a comprehensive Australian methodology for resection, orientation, and rotation analysis in joint replacement surgeries. This protocol has the potential to be used for any type of joint replacement procedure. It utilizes a CT scan acquired prior to surgery and a postoperative CT scan for evaluation in order to generate a computational model of the knee with patient-specific axes in three-dimensional space. The mean effective radiation dosage per CT scan with this approach is 1.24 ± 0.96 mSv [19]. Using the AURORA protocol, patient movement in the CT scan may be detected at any point along the length of the bone. The method showed excellent reliability and reproducibility by removing the sources of error that are typically associated with post-operative total knee arthroplasty analysis. The AURORA protocol involves the creation of a postoperative 3D model, requires recording of both legs, as well as mandatory preoperative recording and long postprocedural processing with the consultation of engineers. The use of special software packages relative to the Perth CT protocol can be applied on any CT scanner.

The United Nations Scientific Committee on the Effects of Atomic Radiation (UNSCEAR) 2020/21 [20] report on medical radiation exposure stated that, worldwide, CT constitutes 9.6% of radiologic examinations and contributes 61.6% of the collective dose. The mean value of the typical effective dose for computer tomography examinations for limbs is 2.1 mSv.

Even though there is no definitive safe level of radiation, it is possible to decrease the effective doses by adjusting and minimizing the dose parameters. This study examined methods for reducing radiation exposure while maintaining image quality for TKA procedures.

## 2. Material and Methods

### 2.1. Study Design and Participants

The cross-sectional study comprised 16 patients who received treatment in a hospital or underwent outpatient examination at the Clinic for Orthopedic Surgery and Traumatology and the Center for Radiology, University Clinical Center of Serbia (UKCS) between May 2021 and September 2022. The participants were provided with a written document with relevant information about the study’s aims and the scope of their rights. Each participant who consented to participate in the study provided a signature. The approval was obtained from the Ethics Committee of the UKCS (Ethical code: 157/7 approved date: 27 April 2021).

Inclusion criteria were individuals with radiographically confirmed severe degenerative osteoarthritis who did not experience any improvement following six months of non-operative therapy (i.e., individuals who required TKA and had not had any previous surgical procedures on the knee). Additionally, the study involved patients who received a postoperative CT scan of the replaced knee prosthesis for routine monitoring purposes, six weeks following the surgery, or if they experienced any concerns. The study was performed in accordance with the guidelines provided by the American College of Radiology (ACR) for imaging following TKA [11].

### 2.2. Protocols of Examinations

All patients were examined on a 160 detector CT scanner (Toshiba, Aqulion Prime Otawara, Japan) according to the Perth CT protocol. The device has the capabilities of automatic exposure control (AEC), sure expose and iterative image reconstruction (IR), adaptive Iterative Dose Reduction 3D (AIDR 3D), as well as the single-energy projection-based metal artifact reduction (MAR) algorithm (SEMAR) technique for eliminating prosthesis artifacts. A scan of the whole leg was performed, in supination and neutral position, from the roof of the acetabulum to the talus arch according to the standard and optimized Perth CT protocol for each patient. Standard Perth CT protocol: scanning with a slice thickness of 0.5 mm i.e., collimation is HP standard 0.5 × 80; pitch factor (PF) 0.813/helical pitch (HP) 65.0; kV are fixed at 120 and mA are fixed at 200 with soft tissue reconstruction (standard soft tissue kernel (FC08)) with 3 mm sections and tube rotation speed 0.5 s. Optimized Perth CT protocol: 0.5 slice thickness with HP fast 0.5 × 80 collimation; PF 1.388/HP 111.0; kV are fixed at 100, and mA are variable (sure expose) and range from 150 to 180 with reconstruction for soft tissues (standard soft tissue kernel (FC08)) at 3 mm and tube rotation speed of 0.5 s (Table 1).

### 2.3. Data Collection

Post-procedural CT measurements were performed on a workstation (Vitrea 2, Vital Images, Minnetonka, MN, USA) with dose reports, i.e., values of CT dose index-volume (CTDIvol (mGy)) and Dose length product (DLP) (mGy × cm). DLP = (CTDIvol) × (length of scan, cm). The effective dose (E) was calculated according to the formula: E = k × DLP. The value of k depends on the observed organ and the age of the patient. According to the manufacturer’s recommendations, it is 0.0008, and its unit is mSv × mGy^−1^ × cm^−1^. The SI unit for effective dose is the Sievert (Sv).

Regular device monitoring was conducted through calibration using a phantom in the air (Quality control—QC test) to ensure and optimize color image quality. The image quality was evaluated using subjective and objective criteria. The subjective criteria involved assessing the visibility of bone structures that are essential for measuring seven alignment measures at three different levels: the femoral head (at the point where the intra-articular ligament attaches), the knee (at the surgical transepicondylar line), and the tibia (at the tibiofibular joint).

The visibility of anatomical structures, specifically the specified bones, is categorized into three grades: grade 1 indicates that details are hardly visible, grade 2 indicates that details are present, and grade 3 indicates that details are clearly visible. These grading criteria are in accordance with the European guidelines on quality criteria for computed tomography, EUR 16262 EN [21]. The identical grading system was utilized to assess the extent of artifacts in individuals with prostheses. The objective assessment of picture quality is based on the calculation of the figure of merit (FOM), which is established by evaluating the ratio of signal difference to noise ratio (SdNR) and the radiation dose (effective dose). A region of interest (ROI) with an area of 10 mm^2^ represents the value of bone tissue in the above-mentioned extremity levels, expressed in Hounsfield units (HU) and related to the I_ROI_. Subsequently, the average value in the background HU (representing soft and muscle tissue) was calculated using the same method like I_ROI_ and realated to the I_BEK._ Then, the value of SdNR was obtained by the following formula: SdNR = (I_ROI_ − I_BEK_)/SD_BEK_, and FOM = SdNR^2^/E was determined. The measurements, both objective and subjective, were conducted for all patients by three radiologists, in two different periods at an interval of two weeks.

### 2.4. Statistical Analysis

Descriptive and analytical statistical methods were used in this study. The results are presented in tables and graphs. Absolute and relative numbers, measures of central tendency (arithmetic mean), and dispersion measures (standard deviation, standard error, and 95% confidence intervals) were used for descriptive purposes. Generalized linear mixed models were used for modeling assessed variables (CTDI, DLP, E, SdNR, FOM) between the Optimized Perth CT protocol (OPT) and the Standard Perth CT protocol (STP). Generalized linear mixed models extend the linear model so that the data are permitted to have a non-normal distribution and the observations can be correlated. The intraclass correlation coefficient (ICC) was used to describe how strongly observations in the same protocol resemble each other between different observers. In all analyses, the significance level was set at 0.05. Statistical analysis was performed using IBM SPSS statistical software (SPSS for Windows, release 25.0, SPSS, Chicago, IL, USA).

## 3. Results

This study included 16 patients (10 (62.5%) females and 6 (37.5%) males), with an average age of 68.75 ± 4.40 years. The average BMI of study population was 29.75 (kg/m^2^), with a maximum value of 36 kg/m^2^ and a minimum value of 24 kg/m^2^, indicating that the majority of patients have excess body weight bordering on obesity.

Imaging was conducted as part of preoperative preparations in 12 patients (75%) who did not have a prosthesis. The other four (25%) patients had a prosthesis, which means that imaging was performed for the purpose of postoperative evaluation. The disease was observed on the right leg in nine individuals, accounting for 56.3% of the cases, and on the left leg in seven individuals, accounting for 43.8% of the cases.

**Figure 1:**
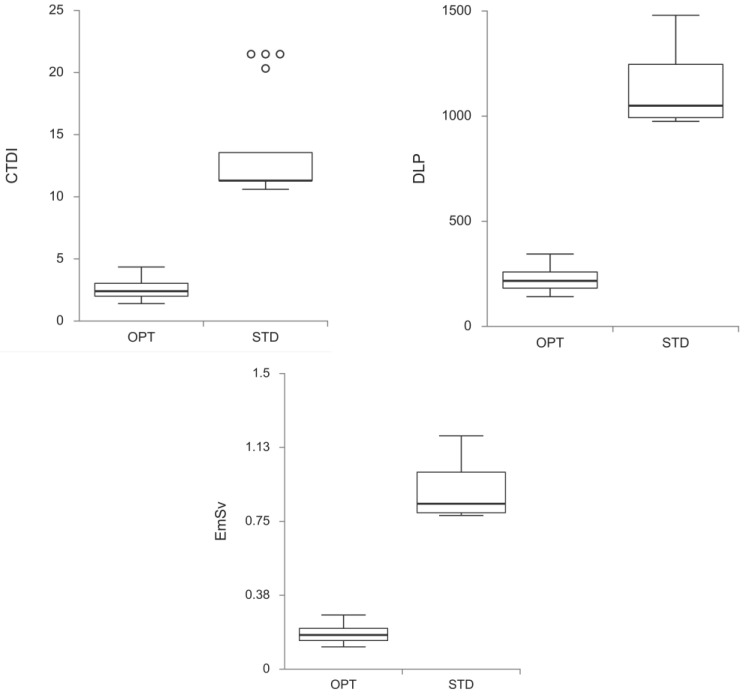
Values of CTDI_vol_, DLP, and effective radiation dose (E) for the optimized protocol (OPT) and the standard protocol (STP); *p* < 0.001 for all. The center line of the box represents the group median, the top and bottom of the box represent the 75th and 25th percentiles. Whiskers are extended to the most extreme data point that is no more than 1.5× interquartile range from the edge of the box (Tukey style). Dots beyond the whiskers represent outliers. CTDI, CT dose index-volume; DLP, dose length product; E, effective dose; OPT, optimized Perth CT protocol; STP, standard Perth CT protocol.

The mean values of CTDI_vol_, DLP, and E were significantly lower when the optimized protocol (OPT) was used, in comparison to the standard protocol (STP) (*p* < 0.001 for all) (Table 2, Figure 1). There was a statistically significant difference between the means of effective radiation dosage for STP and OPT, where the mean value of the effective radiation dose was 0.900 ± 0.034 mSv for STP and 0.180 ± 0.013 mSv for OPT (*p* < 0.001) (Table 2).

**Figure 2:**
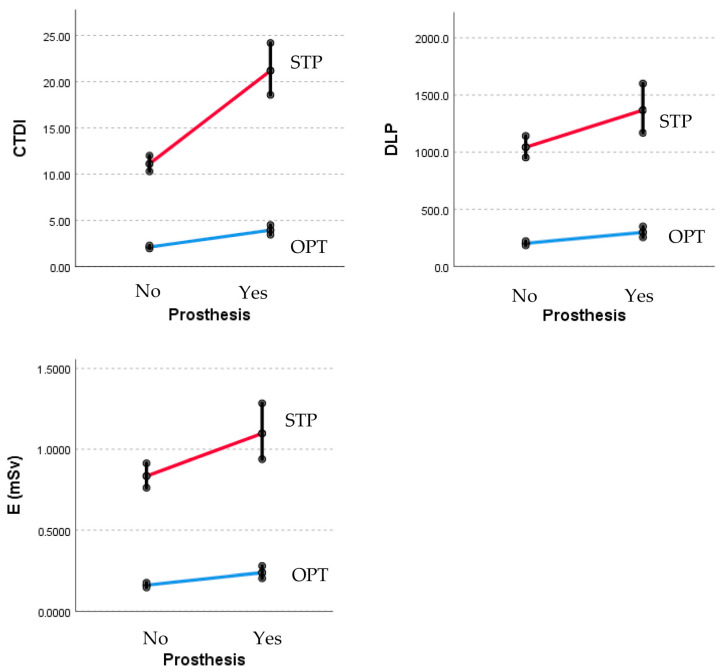
CTDI_vol_, DLP, and E for protocols with/without prosthesis. *p* < 0.001 for all. CTDI_vol_, CT dose index-volume; DLP, dose length product; E, effective dose; OPT, optimized Perth CT protocol; STP, standard Perth CT protocol.

The mean values for CTDI_vol_, DLP, and E are shown in Table 3 and Figure 2. These values are dependent on the existence of a prosthesis and are specific to the two protocols mentioned. The results confirmed that the optimized protocol leads to a lower increase in dosage in patients with prostheses compared to the standard protocol (*p* < 0.001).

Upon analyzing the objective criteria for picture quality, namely SdNR and FOM, it is evident that optimized protocols result in lower SdNR values and higher FOM values (as shown in Table 4). This outcome is predicted, given the manner in which these parameters are obtained.

**Figure 3:**
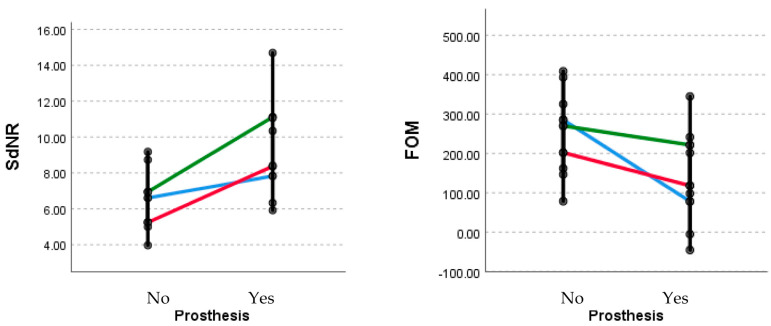
Estimation of image quality determined using the SdNR and FOM as per the specified protocols and parts of extremities; Blue—hip; Red—knee; Green—ankle. *p* > 0.05 for all; FOM, figure of merit; OPT, optimized Perth CT protocol; SdNR, signal difference to noise ratio; STP, standard Perth CT protocol.

The estimation of image quality is determined using the SdNR and FOM as per the specified protocols and parts of extremities, and is presented in Table 5 and Figure 3.

**Figure 4:**
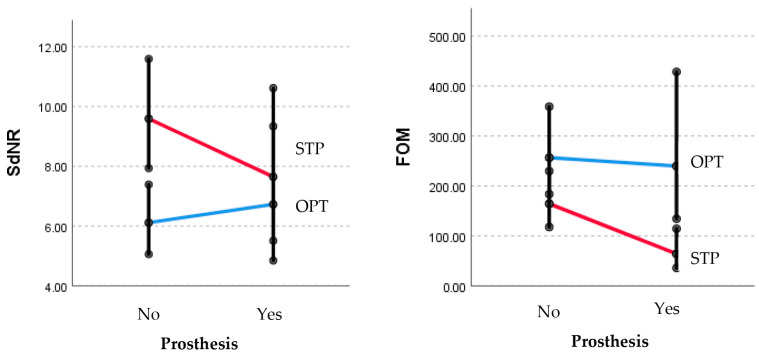
Estimation of image quality determined using the SdNR and FOM as per the specified protocols and the presence of prothesis. *p* > 0.05 for all; FOM, figure of merit; OPT, optimized Perth CT protocol; SdNR, signal difference to noise ratio; STP, standard Perth CT protocol.

The estimation of image quality is determined using the SdNR and FOM as per the specified protocols and the presence of prothesis, and it is presented in Table 6 and Figure 4.

No significant difference is observed in the subjective evaluation of image quality between protocols (*p* > 0.05). Table 7 shows the subjectively evaluated visual quality using techniques tailored to different viewers. These is a moderate to high degree of agreement between viewers on both protocols for STD protocol: ICC from 0.750 (knee and ankle) to 0.932 (knee) and for OPT protocol: ICC from 0.655 (knee and ankle) to 0.933 (knee).

## 4. Discussion

This study showed that when performing the Perth CT protocol, by reducing the kV and mAs values, as well as by changing the collimation and increasing the pitch factor, the effective dose of radiation can be reduced five times and the image quality will be satisfactory. Advanced iterative reconstruction (IR) techniques were used in both protocols, and automatic exposure control (AEC) was used in the optimized protocol.

Our study showed that the effective radiation dose with the STD protocol is lower than Perth CT [13] and AURORA [19], and slightly higher than the Imperial protocol [18] and long-leg standard radiography [17], and significantly lower with the OPT protocol.

In this research, a 160-slice CT scanner was used, which enables faster acquisition and thinner sections, while 4.16 and 64-slice CT scanner was used in the work of Henckel at al. [18]. Technical parameters that directly affect the dose and image quality are [22]: nominal slice thickness, inter-slice distance/pitch factor, volume of investigation, and exposure factors related to the properties of the X-ray tube: voltage (kV), current (mA), and exposure time (s), then field of view (FOV), gantry tilt, reconstruction matrix, reconstruction algorithm (filter, kernel), window width and window level.

Two important scanning parameters affecting the radiation exposure in CT are tube current and tube voltage [22,23]. Increasing the voltage exponentially increases the radiation dose, while lower voltage means better contrast. The usual value of 120 kV is rarely changed. Reducing the current strength directly proportional decreases the radiation dose, but increases the image noise, which can affect the diagnostic outcome of the examination. Modern scanners have gentry rotation times in the region of 0.4 s. The main consequence of reducing the rotation time is an increase in noise and a reduction in the absorbed dose. The pitch factor has a direct influence on patient radiation dose; as pitch increases, the time that any one point in space spends in the X-ray beam is decreased.

In their study, Henckel et al. [18] reduced the effective dose by reducing volume of investigation, i.e., they performed a separate imaging of the leg at the level of region of interest for adequate measurements: 5 cm at the hip level, 20 cm at the knee level and 5 cm at the ankle level; kV was fixed at 120, and mAs were corrected depending on the region (hip level: 80, knee level: 100, ankle level: 45), and collimation was reduced depending on the region of imaging and the number of CT machine detectors and thus obtained a significant dose reduction. A reduction in dose was noted at 64 slices compared to 4 and 16 slices in CT scanners due to collimation [18]. The same was done in this study. KV, mAs were reduced, the pitch factor was increased, but the entire leg was recorded. Separate imaging requires additional software tools for image reconstruction and as such cannot be applied to all CT scanners.

In a study by Chauhan et al. [13], which was conducted on cadavers, the CT protocol involved a scan sequence that was performed from the superior margin of the acetabulum to the talus, using 2.5 mm contiguous slices. The scan time was 40 s with an average kilo-voltage of 140 and 85 mA. The calculation radiation dose for the procedure was 2.5 mSv, which could have been reduced to 1 mSv by the use a lead shield [13].

Huppertz et al. [24] compared radiation exposure and image quality between dedicated computed tomography protocols for preoperative total hip arthroplasty (THA) planning. Three protocols with automated tube current modulation using 64-slice and 128-slice CT scanners without and with automated tube voltage preselection (of 120 and 100 kV) were compared. In groups without automated tube voltage preselection, kV was fixed at 120. In all patients, the tube current was automatically modulated. In our study, we used AEC only for the optimized protocol. The tube voltage allowing for the lowest radiation exposure while keeping the desired level of noise is then automatically selected. Iterative reconstruction algorithms were not applied in the study, while we used IR for both protocols. In their study, mean effective dose was 2.8 mSv. Lowest radiation exposure (2.5 mSv) was seen with automated voltage preselection, and the algorithm’s selection was 100 kV (90.5% of patients) and 120 kV. Lowest image noise was seen in the highest dose group (3.1 mSv, 128-slice CT fixed tube voltage). They concluded that preoperative pelvic CT for THA planning is possible with very low radiation dose and reliable quality. Automated voltage preselection further decreases the effective dose by 18.2%.

In order to reduce radiation dose, many technical innovations have been developed, such as automatic exposure control and iterative image reconstruction [25]. The CT scanner on which this research was done has these capabilities and thus leads to lower values of effective dose compared to other methods. These are factors that indirectly affect the dosage, and directly affect the quality of the image.

Automatic exposure control is one of the most important aspects of radiation dose and image quality optimization for CT scanning. It refers to automatic adaptation of mA on the basis of user-specified image quality and X-ray attenuation characteristics of the scanned body region [26]. Most modern multiple-detector-row CT scanners use up to three major types of spatial mA modulation techniques (*x*-*y* axis/angular; *z* axis/longitudinal; *x*-*y*-*z*/combined). AEC as an advanced CT technique is called differently depending on the vendors. Greess and al. [27] showed a dose reduction of 39% in the knee region with attenuation-based on-line modulation of the tube current. In our study, the tube current was automatically modulated (Sure expose) in the optimized protocol, which certainly contributed to lowering the radiation dose. However, the kV value can also be automatically selected prior to the CT scan when the Automatic Tube Voltage Selection (ATVS) feature is available [28]. Nakayama et al. [29] are done and McColloug [30] citated this study in which performed abdominal CT in 40 patients at 120 kV, with follow-up scanning performed at 90 kV. Although they did not increase the tube current–time product at the lower tube voltage setting, overall image quality and enhancement of abdominal organs were not significantly different between the examinations performed at 120 kV and at 90 kV (*p* > 0.05). What was different was dose, with a weighted CTDI reduction of 56.8% at the lower tube voltage.

Iterative reconstruction techniques [31], as the name suggests, iterate the image reconstruction several times to better estimate these mathematics assumptions and generate images with lower noise. Different scanner manufacturers have taken different algorithmic approaches to iterate different components of the image reconstruction algorithm. However, the common endpoint of all the iterative reconstruction algorithms is to produce lower image noise and higher resolution by maintaining edges and lower artifacts. This ability of iterative reconstruction techniques enables use of reduced-dose CT with lowering of scanning parameters, such as tube current or even tube potential. It is given different names depending on the vendors.

Manufacturers claim that the dose is reduced by 80% with iterative reconstruction (IR) [32], but independent studies show significant reduction ranging from 32 to 65% [33]. Deák et al. [34] performed subjective and objective image quality assessment of abdominal CT images with model-based IR (MBIR, GE Healthcare) and reported that use of MBIR algorithm decreased image noise by up to 47% compared with adaptive statistical iterative reconstruction (ASIR) and 58% compared with non-iterative filtered back projection (FBP) algorithms.

The optimized protocol from our study showed a lower effective dose of radiation than conventional long-leg radiography. Whole-leg scanning has its advantages. Abu-Rajab et al. [35] showed that hip–knee–ankle radiography is more appropriate for assessment of post-operative mechanical alignment of total knee arthroplasties than standard AP knee radiography. Long-leg radiography allows weight-bearing imaging. CT has proven to be extremely helpful for evaluation of patients with painful TKA [11,36], and advances in multi-detector CT have improved the image quality by further minimizing metal artifacts. Review of a large number of CT scans of painful TKA with equivocal plain film has shown this modality to be particularly effective for the following indications: loosing, osteolysis, assessment of rotational aliment of the femoral component relative to the transepicondylar axis and detection of subtle or occult periprosthetic fracture.

Cehic et al. [37] compared mechanical alignment in long-leg radiography and Perth CT protocol before and postoperatively after TKA. They showed that there is excellent agreement of results preoperative and good agreement postoperatively, but that due to other information obtained by CT examination, it is advised that all patients in whom the planned elective TKA is performed be given a preoperative CT scan. The effective radiation dose of their Perth protocol was 1.43 mSv.

In postoperative evaluation, the knee prosthesis creates metal artifacts. Various methods have been introduced to reduce the metal artifacts, including higher peak voltage, higher tube charge, MAR algorithms and dual-energy CT techniques [38,39]. However, higher peak voltage and tube charge may only reduce metal artifacts to a minor degree and may lead to a higher radiation dose for the patient [40]. Therefore, CT with metal artifact reduction (MAR) and dual-energy techniques are currently used to reduce the metal artifacts. Gondim Teixeira et al. [41] concluded that CT images with IR alone yielded poor visualization of the periprosthetic soft tissues in patients with hip arthroplasty. The association of IR with the SEMAR algorithm led to significant improvement in quality, even in patients with bilateral prostheses. Zhang at al. [40] showed that the SEMAR algorithm plus IR can significantly reduce metal artifacts and increase diagnostic confidence of prosthetic complications and tumor recurrence in patients with knee tumor prostheses than IR alone.

In our research, the SEMAR plus IR algorithm was applied to reduce metal artifacts from knee prostheses, and the quality of the images according to subjective criteria was satisfactory in both the standard and the optimized protocol in patients with knee prostheses. Also, there was no significant difference between the assessors in the subjective criteria for image quality. Interestingly, SdNR in patients with knee prostheses in the optimized protocol was larger compared to those without prostheses, which can be explained by using the SEMAR technique. With respect to 320-row CT scanners, Kidoh et al. [42] showed that SEMAR is a better metal-artifact-reduction strategy for CT venography after knee replacement surgery than dual-energy CT-based monochromatic imaging. The mean SdNR was significantly higher on the MAR than the monochromatic images, and the visual scores were significantly higher for MAR than monochromatic images. In their study, the total CTDI for dual energy scanning was 27.7 mGy. By adopting the 80 kVp  +  SEMAR technique, the radiation dose can be reduced by 42%.

In a study with cadavers, on a 320-detectror row CT scanner (Aquilion ONE, Canon Medical Systems, Otawara, Japan), Barreto et al. [43] showed that SEMAR was more effective at reducing metal artifacts than dual-energy CT (DECT). The radiation dose in cadavers with TKA was lower in the application of SEMAR compared to DECT (CTDI_vol_ was 14.6 vs. 15.8 mGy). AEC was applied to all [43].

Recently, Chun et al. [44] presented a novel image quality evaluation method that allows a fully automated assessment of three image quality metrics (noise level, structure sharpness, and alteration of structure) on patient CT images. They applied this method to the contrast-enhanced liver CT images from four different CT scanners reconstructed with filtered back projection (FBP), vendor-specific iterative reconstruction (IR), and a vendor-agnostic deep learning model (DLM). DLM showed statistically superior performance to IR in all three image quality metrics. This study is expected to contribute to enhance the CT protocol optimization process by allowing a high throughput and quantitative image quality evaluation during the introduction or adjustment of lower-dose CT protocol into routine practice.

In our research, there was no statistically significant difference in SdNR and FOM values depending on the measurement site (hip, knee, ankle), which indicates that this optimization principle can be applied to any part of the limb.

Moldovan et al. [45] showed that the COVID-19 pandemic severely affected the volume of arthroplasty performed in 120 hospitals in Romania (decrease of up to 55.47% for primary interventions and 69.14% for revision interventions in hip and knee arthroplasty). They pointed out change guidelines specific to orthopedic surgery during this critical period. They proposed the development of new procedures and alternative clinical solutions for possible future outbreaks. Our study can be one of these solutions.

Limitations of the research include the small sample size and a small number of patients with knee prostheses, but the results show that it is justified to do research on a larger number of patients as well as with other prostheses.

## 5. Conclusions

Retrospecting the ALARA (As Low As Reasonably Achievable) principles, it is possible to optimize the Perth CT protocol, first by changing the basic parameters of the examination protocol that directly affect the radiation dose (kV, mA, pitch factor, collimation, research volume) and also by using advanced techniques such as IR and AEC that indirectly affect the dose and directly affect the image quality. The effective dose of radiation can be reduced five times, and the image quality will remain satisfactory. The aim of our study was to understand the importance of optimizing CT protocols (the effective dose of radiation is reduced five times and the image quality will still be satisfactory). We have shown that the increase in the effective radiation dose in patient with a knee prosthesis by optimized protocol was significantly lower compared to those with a standard protocol and that the image quality according to objective criteria (SdNR) was discretely better in patients with a knee prosthesis by optimized protocol compared to those without a knee prosthesis primarily due to the SEMAR technique. Limitations of the research include the small number of patients with prostheses.

## Figures and Tables

**Table 1:** Schematic representation of the acquisition parameters for protocols.

	Standard Perth CT Protocol	Optimized Perth CT Protocol
kV	120	100
mA	200	Sure expose (150–180)
Collimation	HP standard 0.5 × 0.8	HP fast 0.5 × 0.8
Pitch factor	0.813/HP 65	1.388/HP 111
SEMAR	+	+
Slice thickness	0.5 mm	0.5 mm
Reconstruction	3 mm	3 mm
Rotation time	0.5 s	0.5 s
Kernel	FC08	FC08

**Table 2:** Radiation according to protocols.

	OPT Mean ± SE 95% CI for Mean	STP Mean ± SE 95% CI for Mean	Mean Difference ± SE 95% CI for Mean Difference	*p*
CTDI_vol_ (mGy)	2.58 ± 0.23 (2.12–3.04)	13.64 ± 1.13 (11.33–15.95)	11.06 ± 1.15 (8.71–13.41)	<0.001
DLP (mGy·cm)	225 ± 16 (193–257)	1123 ± 42 (1037–1210)	898.59 ± 45.02 (806.65–990.54)	<0.001
E (Sv)	0.180 ± 0.013 (0.155–0.206)	0.900 ± 0.034 (0.830–0.969)	0.72 ± 0.04 (0.65–0.79)	<0.001

CI, confidence interval; CTDI, CT dose index-volume; DLP, dose length product; E, effective dose OPT, optimized Perth CT protocol; SE, standard error; STP, standard Perth CT protocol.

**Table 3:** Radiation according to protocols with/without prothesis.

		OPT	STP
CDTI_vol_ (mGy)	Without prothesis	2.13 ± 0.08 (1.97–2.29)	11.13 ± 0.42 (10.30–12.00)
	With prothesis	3.95 ± 0.26 (3.46–4.50) *	21.19 ± 1.40 (18.57–24.19)
DLP (mGy·cm)	Without prothesis	201 ± 9 (1.97–2.29)	1043 ± 46 (952–1042)
	With prothesis	297 ± 22.8 (254–348) *	1367 ± 104 (1168–1600)
E (Sv)	Without prothesis	0.161 ± 0.007 (0.147–0.176)	0.834 ± 0.037 (0.761–0.913)
	With prothesis	0.238 ± 0.018 (0.203–0.278) *	1.098 ± 0.084 (0.938–1.284)

* *p* < 0.001; CTDI_vol_, CT dose index-volume; DLP, dose length product; E, effective dose; OPT, optimized Perth CT protocol; STP, standard Perth CT protocol.

**Table 4:** Image quality SdNR for FOM according to protocols, on all patients, independent of parts of extremities.

	OPT	STP	*p*
SdNR	6.22 ± 0.50 (5.20–7.31)	9.00 ± 0.73 (7.66–10.57)	0.002
FOM	252 ± 36 (181–324)	139 ± 36 (68–210)	0.028

FOM, figure of merit; OPT, optimized Perth CT protocol; SdNR, signal difference to noise ratio; STP, standard Perth CT protocol.

**Table 5:** Estimation of image quality determined using the SdNR and FOM as per the specified protocols and parts of extremities.

		OPT	STP
SdNR	Hip	6.61 ± 0.93 (5.83–8.73)	7.83 ± 1.10 (7.66–10.57)
	Knee	5.24 ± 0.74 (4.00–6.92)	8.37 ± 1.18 (3.33–11.00)
	Ankle	6.94 ± 0.98 (5.25–9.18)	11.16 ± 1.56 (8.41–14.69)
FOM	Hip	285 ± 62 (162–409)	78 ± 61 (−45–201)
	Knee	202 ± 62 (79–325)	118 ± 62 (−5–242)
	Ankle	270 ± 62 (147–393)	221 ± 62 (98–345)

*p* > 0.05 for all; FOM, figure of merit; OPT, optimized Perth CT protocol; SdNR, signal difference to noise ratio; STP, standard Perth CT protocol.

**Table 6:** Estimation of image quality determined using the SdNR and FOM as per the specified protocols and the presence of prothesis.

		OPT	STP
SdNR	Without prothesis	6.11 ± 0.58 (5.05–7.39)	9.59 ± 0.92 (7.93–11.59)
	With prothesis	6.72 ± 1.11 (4.84–9.37)	7.64 ± 1.26 (5.50–10.62)
FOM	Without prothesis	256 ± 43 (184–359)	164 ± 28 (118–230)
	With prothesis	240 ± 70 (134–429)	64 ± 19 (36–114)

*p* > 0.05 for all; FOM, figure of merit; OPT, optimized Perth CT protocol; SdNR, signal difference to noise ratio; STP, standard Perth CT protocol.

**Table 7:** Subjectively assessed image quality on both protocols.

Viewer	Image Quality	STP (*n*)			OPT (*n*)		
		Hip	Knee	Ankle	Hip	Knee	Ankle
Viewer 1	Hardly visible	0	0	0	0	0	0
	Present	0	4	0	0	4	0
	Clearly visible	16	12	16	16	12	16
Viewer 2	Hardly visible	0	2	0	0	2	0
	Present	2	4	2	3	4	3
	Clearly visible	14	10	14	13	10	13
Viewer 3	Hardly visible	0	1	0	0	1	0
	Present	2	3	2	2	4	2
	Clearly visible	14	12	14	14	11	14

OPT, optimized Perth CT protocol; STP, standard Perth CT protocol.

## Data Availability

Data are available from the corresponding author upon reasonable request.

## Abbreviations

TKA	Total knee arthroplasty
US	United States
AURORA	Australian Universal Resection Orientation and Rotation Analysis
UNSCEAR	United Nations Scientific Committee on the Effects of Atomic Radiation
mSv	Millisievert
UKCS	University Clinical Center of Serbia
ACR	American College of Radiology
AEC	Automatic exposure control
IR	Iterative reconstruction
SEMAR	Single-energy projection-based MAR (metal artifact reduction)
MAR	metal artifact reduction
PF	pitch factor
HP	helical pitch
CTDI_vol_	CT dose index-volume
mGy	Milligray
DLP	Dose length product
Cm	Centimeter
E	Effective dose
Sv	Sievert
QC	Quality control
FOM	Figure of merit
SdNR	Signal difference to noise ratio
ROI	Region of interest
HU	Hounsfield units
I	Intensity
OPT	Optimized Perth CT protocol
STP	Standard Perth CT protocol
ICC	Intraclass correlation coefficient
FOV	Field of view
THA	Total hip arthroplasty
MBIR	Model-based iterative reconstruction
ASIR	Adaptive statistical iterative reconstruction
FBP	Filtered back projection
DLM	Deep learning model
ALARA	As Low As Reasonably Achievable
